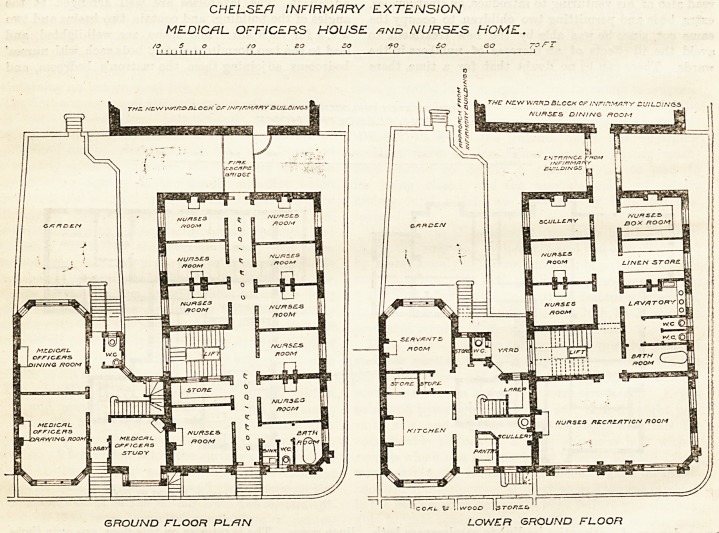# Hospital Construction

**Published:** 1898-01-08

**Authors:** 


### HOSPITAL CONSTRUCTION.

### ST. ANNE'S-ON-SEA CONVALESCENT HOME.

We referred to the opening of this home in our issue of November 27th, and now publish the plans. The building accommodates 12 boys and 12 girls, sent there from the Manchester Children's Hospital, and on the ground floor there are two play-rooms, facing south and west, one 20 ft. by 18 ft., and the other 5 ft. longer. Adjoining the latter is the joint dining-room, 20 ft. by 18 ft., and beyond this, on the north side, are the kitchen, scullery, pantry, &c. The entrance is on the east, and the waiting-room and matron's sitting-room adjoin it. The lavatories are well arranged at the angles of the building, and contain two basins and two w.c.'s for each sex. The stairs are well-lighted, and lead to the two dormitories of 12 beds each, with nurses' bedrooms adjoining them, the matron's bedroom, and

GROUND FLOOR PLAN

FIRST FLOOR PLAN

linen-room. The lavatories on this floor are over those below, and contain a slop-sink, bath, three basins, and w.c. for each sex. The dormitories are lofty, and extend, with dormers to assist in their lighting and ventilation, into the roof, which also contains accommodation for the servants. The principal rooms are warmed by hot water, but those on the ground floor have fire-places as well. The plan appears to us to be, for the number of patients accommodated, an economical and satisfactory one, and it is compact, without sacrificing good lighting and ventilation, as too often happens in buildings of this class. The architects are Messrs. Salomons and Steinthal, of Manchester.

### NURSES' HOME, CHELSEA INFIRMARY.

The plans show the nurses' home and medical officer's house, erected from the designs of Messrs. Lansdell and Harrison, in connection with the extension of the infirmary. The house has several good features in its plan, and contains three sitting-rooms and five bed-rooms on three floors, with kitchen, &c, in a well-lighted lower ground floor. Two floors of the home extend over its top storey, but it is, of course, self-contained and entirely separate.

The lowest floor of the home contains a recreation-room 32 ft. by 18 ft., at the corner of Sydney Street, facing south and west, with w.c.'s, bath-room, lavatory, linen store, and box-room adjoining. Across the passage are two nurses' rooms and a small kitchen, and the remaining space is occupied by the staircase, in the well of which there is a lift for coals, &c.

give the superintendent control of the lighting. The stairs, corridors, and recreation-room are warmed by steam from the infirmary. The planning throughout appears to be admirable, and the only criticism we would make is that the accommodation appears to be almost too good, the rooms being rather larger than necessary and the lavatory accommodation a little too lavish. In spite of governmental control, there is a tendency for rate-supported institutions to out-do the efforts of the best voluntary hospitals, pressing the latter somewhat hardly in the race for improvement.

CHELSEA INFIRMARY EXTENSION
MEDICAL OFFICERS HOUSE AND NURSES HOME.

GROUND FLOOR PLAN LOWER GROUND FLOOR

The ground floor plan is repeated on the four floors above, except that two of them extend over the medical officer's house, and contain separate bed-rooms for 42 day nurses and 13 night nurses, the latter being shut off from the stairs so as to secure the necessary quiet. The rooms are generally about 13 ft. by 9 ft. by 10 ft. high, the superintendent and charge nurses having slightly larger apartments. Every room has a fire-place, and special arrangements for ventilation. On the third floor there is a sitting-room and kitchen for the night nurses, and the lavatory accommodation (w.c., slop-sink, and bath) is repeated on every floor. Besides the use of fire-proof floors, and a system of hydrants, safety is further provided for by access to the iron staircase on the north, which is also available for the inmates of the infirmary. The building is lighted throughout by electricity, with gas as a reserve, and the switches are arranged so as to prevent waste and to

## Figures and Tables

**Figure f1:**
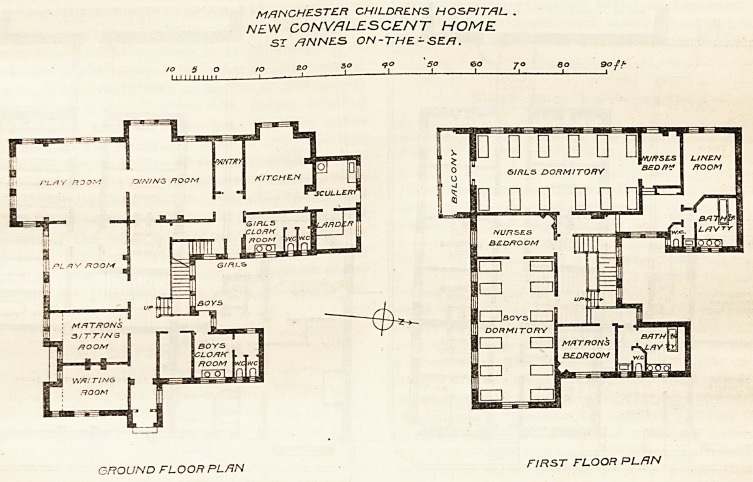


**Figure f2:**